# Adaptation and Implementation of an Intervention Programme on Spanish Carers and Adolescent Patients With an Eating Disorder: Study Protocol of a Randomized Controlled Trial

**DOI:** 10.3389/fpsyg.2021.697916

**Published:** 2021-10-22

**Authors:** Yolanda Quiles, María José Quiles, Eva María León, María Roncero, Álvaro Ruiz, Maite España, Cristina Romero, Vicente Elvira

**Affiliations:** ^1^Department of Behavioral Sciences and Health, University Miguel Hernández, Elche, Spain; ^2^Department of Personality, Assessment and Psychological Treatments, University of Valencia, Valencia, Spain; ^3^Department of Personality, Assessment and Psychological Treatment, University of Murcia, Murcia, Spain; ^4^Unit of Eating Disorders, University Hospital of San Juan de Alicante, Alicante, Spain

**Keywords:** eating disorders, adolescents, skills sharing, carer skills, randomized controlled trial

## Abstract

**Introduction:** One of the major problems with inpatient treatment of adolescent girls with an eating disorder (ED) is that the strategies learned during their hospital stay are not easily applied or maintained in their daily lives, and this has been related to high rates of relapse and readmission. The ECHOMANTRA programme was developed to optimize outcomes during and following inpatient or day-patient treatment. ECHOMANTRA is based on interventions for carers (Experienced Carers Helping Others, ECHO) and patients (Maudsley Model of Anorexia Nervosa Treatment for Adults, MANTRA) and is developed from the cognitive interpersonal model of anorexia (Schmidt and Treasure, 2006; Treasure and Schmidt, 2013). This study aims to describe the study protocol of a randomized controlled trial (RCT) for evaluating the efficacy of an adaptation of a novel intervention for patients and carers (ECHOMANTRA) to be implemented as an add-on to treatment-as-usual (TAU).

**Method:** In a multi−center pilot RCT, 80 female adolescent patients with a DSM-5 diagnosis of an ED and their carers will be invited to participate in the study. They will then be randomized to receive either the ECHOMANTRA intervention as an add-on to TAU or TAU alone. A repeated measures design will be conducted across four time points. Primary outcomes will be patient psychological well-being and eating disorder symptoms, and secondary outcomes will include body mass index, obsessive-compulsive symptoms, perfectionism, motivation to change and psychosocial adjustment. For carers, outcome variables will include psychological well-being, expressed emotion, accommodation and enabling behaviors, burden, and care skills.

**Discussion:** The results from this trial will establish the effectiveness of ECHOMANTRA and may reveal whether and to what extent this novel intervention can optimize outcomes during and following inpatient treatment. This study will also provide the adaptation of the ECHOMANTRA in the Spanish context for inpatient/day-care treatment.

## Introduction

Eating disorders (ED) are extremely complex multi-causal mental health illnesses, which have serious medical complications and especially affect adolescents and young women (López and Treasure, 2011). The most frequent diagnosis in adolescents is Other Specified Feeding and Eating Disorder (OSFED) followed by Anorexia Nervosa (AN) and finally Bulimia Nervosa (BN) (Swanson et al., 2011). These disorders are characterized by serious symptoms as well as a high degree of comorbidity and mortality (Nordbo et al., 2012; Saldaña et al., 2014; Fichter and Quadflieg, 2016); consequently, patients need to be hospitalized on many occasions. One of the major problems with hospital treatment is that although these patients learn strategies during their hospital stay, they have difficulty in being able to apply and maintain them in their daily lives. As a result, there is a high rate of relapses and readmissions, which have been related to resistance to treatment, low motivation to change, severe pretreatment caloric restriction, low body mass index and higher occupational and social stress (Fairburn, 2008; Kaplan et al., 2009; Grilo et al., 2012; Hoang et al., 2014; Morris et al., 2015; Vall and Wade, 2015). A recent meta-analysis study found that the risk of relapse is especially high during the first year after the end of treatment (Berends et al., 2018; Khalsa et al., 2017). High relapse rates reveal the need to optimize patient treatments after hospital admissions by supporting inpatient/day care and community transition through promising targeted psychological interventions (Bryan et al., 2021).

In response to this, Dr. Janet Treasure’s team developed the ECHOMANTRA intervention programme, as a means of facilitating the transition from inpatient hospital treatment to daily life and to the community (Treasure and Schmidt, 2013; Treasure et al., 2015; Cardi et al., 2017). This programme is based on scientific evidence that demonstrates that interventions directed at these patients as well as their carers improve the outcomes in these patients’ health. Involving the family in the treatment of ED is a key strategy in their recovery and it also strengthens patients’ social networks and eliminates their isolation and loneliness, which contribute to maintaining the disorder (Levine, 2012; Treasure and Nazar, 2016; Sepúlveda et al., 2020). A study was recently published with the preliminary results of ECHOMANTRA intervention with AN inpatients and their carers in the United Kingdom (Adamson et al., 2019). A case series study was employed using a mixed-methods approach to measure the feasibility and efficacy of augmenting intensive hospital treatment with ECHOMANTRA. Results showed that patients’ symptomatology improved after the ECHOMANTRA intervention and was maintained in the follow-up. In addition, there was a reduction in carer burden and an improvement in skills, so it was concluded that the efficiency of hospital attention for ED can be increased by preparing both patients and their carers for the transition after hospitalization.

ECHOMANTRA consists of an intervention programme for ED carers (ECHO; Experienced Carers Helping Others; Treasure et al., 2015) and another programme for patients (MANTRA, Schmidt et al., 2014). MANTRA is recommended for the treatment of adults outpatients with AN (National Institute for Health and Care Excellence [NICE], 2017). It is based on the Cognitive Interpersonal Maintenance model of AN (Schmidt et al., 2014), which was developed as a proposed theoretical framework that would synthesize the most important internal and interpersonal maintaining factors of this disorder. MANTRA intervenes in the emotional regulation and eating behavior of these patients, putting a special emphasis on behavior change strategies. This programme focuses on the steps that will help patients thrive in their transition from inpatient care to daily life as well as generating cognitive and behavior changes and strengthening relationships with their family and social groups. It is a flexible treatment programme which directly involves patients in the therapeutic process. To date, it has not been applied to adolescents with an ED; however, some of its characteristics might be especially beneficial to them. In fact, it has been published the study protocol of a recent research that analyses the feasibility, acceptability and efficacy of the MANTRA treatment programme for adolescents patients (Wittek et al., 2021). MANTRA includes content that is prevalent and important to address across the spectrum of ED and in adolescent patients.

The ECHO part of the intervention focuses on carers. Based on the Model of Carer Coping (Treasure et al., 2003), this part of the programme reveals the different aspects that influence coping which can be problematic for carers as they represent sources of psychological distress. The programme provides assistance, support and training for carers to enable them to cope with their role. It teaches them how to reduce and manage their expressed emotion and symptom accommodation, and to deal with difficult and problematic situations that arise. In addition, ECHO teaches skills for positive communication and behavior change so carers will be able to support their loved ones in their recovery. Different studies using the ECHO have shown that the intervention in both the adult and adolescent groups led to a moderate reduction in time spent caring and also in bed use. Moreover, through this programme there was a small to moderate improvement in the wellbeing of both carers and patients in the intervention group (Hibbs et al., 2015a; Magill et al., 2016; Hodsoll et al., 2017). Our research group applied an intervention programme based on the ECHO to carers of ED patients in Spain. The results obtained showed that carers who participated in the programme improved their levels of well-being, reducing carer burden, psychological distress, and expressed emotion. In addition, only patients whose carers participated in the programme reduced their levels of anxiety, depression, and psychological distress while no change occurred in patients whose carers participated in the control group (Pérez-Pareja et al., 2014; Quiles Marcos et al., 2018). A recent review study analyzing the effectiveness of treatments for carers of ED patients concluded that ECHO also provides an intervention that can reduce service costs (Treasure et al., 2021).

The aim of this paper is to describe the study protocol of a randomized control trial (RCT) aimed at evaluating the efficacy of a novel intervention for patients and carers, called ECHOMANTRA, adapted to be used as an add-on to treatment-as-usual (TAU; inpatient treatment or intensive day-care treatment) compared to TAU alone.

### Hypotheses

• Patients from the experimental group (TAU + ECHOMANTRA) will show significantly greater improvements in health outcomes (body mass index, ED symptoms, psychological well-being, psychosocial adjustment, perfectionism, obsessive-compulsive symptoms, motivation to change), and other efficacy indicators as readmission, in comparison to patients from the control group.
• The efficacy of the combined intervention (TAU + ECHOMANTRA) will be stable in the short (6 months) and middle term (9 months).
• Carers from the experimental group will present a better psychological well-being and lower illness accommodation, expressed emotion and burden in comparison to carers from the control group.
• Carers from the experimental group will have more ED carer skills in comparison to carers from the control group.

## Methods and Analyses

This study has been registered on the ISRCTN registry (Trial Identifier: ISRCTN43554732). CONSORT 2010 for parallel group randomized trials (Schulz et al., 2010) is specifically observed in reporting this trial.

### Study Design and Procedure

**FIGURE 1:**
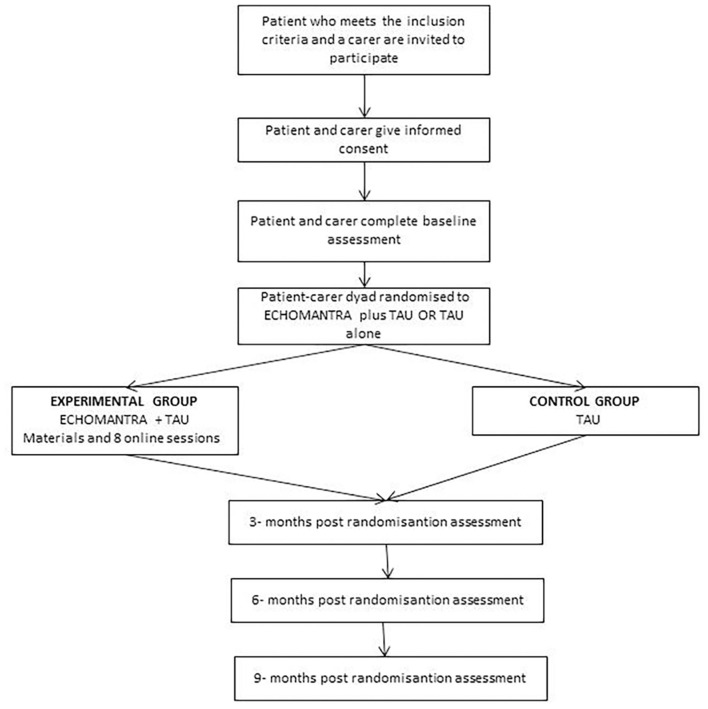
Study design flow diagram.

This is a multi-center, pilot, randomized, controlled, blind, superiority study with two parallels groups. Assignment to the control or experimental group will carry out using a computer-generated randomized sequence, with1:1 treatment allocation. The research assistant at each center will conduct a semi-structured interview to evaluate participants and confirm fulfillment of the inclusion/exclusion criteria. Patients who are receiving ED treatment (either as inpatients or day-patients) and fulfill the inclusion criteria, will be invited to participate in the study together with a carer. By “carer” we refer to someone who usually takes care of the patient outside the hospital/day-center and lives with her. Patients and carers will receive detailed information on the study and will be asked for a written informed consent to be able to participate. After submitting the consent form, participants will be invited to complete the baseline questionnaires and will then be randomly assigned to either (1) ECHOMANTRA in addition to TAU or (2) TAU only (see Figure 1). The ECHOMANTRA-guided skills-sharing intervention will include materials and eight online sessions (one per week) for carers and patients, while treatment duration will be 8 weeks.

### Participants

#### Patients

Patients will be recruited from three different specialist inpatient/day-patient eating disorder units (Eating Disorders Inpatient Unit at the San Juan Hospital; CREA, Centre for emotional and nutritional recovery, Eating Disorders Day Centre; ADANER, Association for the defense of AN and BN care, Eating Disorders Day Centre). This study will involve adolescent girls who have received an eating disorder diagnosis according to the fifth edition of the Diagnostic and Statistical Manual of Mental Disorders (DSM-5) criteria (American Psychiatric Association, 2013), including diagnoses of AN, BN, and OSFED. The inclusion criteria will be as follows: (1) aged between 12 and 19, (2) no psychiatric comorbidity, (3) receiving treatment for ED in a specialist inpatient/day-patient ED unit, (4) a family member willing to participate in the study and (5) ability to manage an electronic device (e.g., mobile phone, computer, laptop or tablet) and the Internet in order to access the online sessions, (6) a native Spanish speaker or understands native level Spanish. Patients will be asked to nominate a carer to whom the researcher will invite to participate in the trial. One carer per patient will be permitted, and this should be a primary carer, somebody who usually takes care of the patient outside the hospital/day-center and lives with her.

#### Carers

For carers/family members the inclusion criteria will be as follows: (1) living with the patient and (2) not suffering from a serious medical or psychiatric condition (this information will be assessed through a self-report) and (3) ability to access an electronic device (e.g., mobile phone, computer, laptop or tablet) and the Internet in order to access the online sessions and (4) a native Spanish speaker or understands native level Spanish.

### Randomization

After screening and consent, patients and their carers will be randomized as a dyad using a randomized computer-generated sequence. A full randomization procedure macro will be applied saving the “seed” (SEED = 13012021) to reproduce the exact selection. Randomization will be facilitated by S. L., a colleague from the Behavioral Sciences and Health Department, who will be not involved in this study. Once the allocation has been assigned, no changes can be made. Those randomized to the intervention arm (ECHOMANTRA plus TAU) will have access to the intervention materials.

### Sample Size

An intention-to-treat method will be used to calculate sample size, considering the hypotheses for the primary outcome variables. Power calculations will be based on power determination for longitudinal designs with attrition (Hedeker et al., 1999), an alpha of 0.05, four assessment points (with baseline serving as a covariate), dropout rate of 40% and a fixed autoregressive coefficient of 0.40. A group size of 40 patients per group would provide 80% power with a bilateral *p* < 0.05 to detect a clinically significant change in eating pathology (overall Eating Disorder Examination Questionnaire, EDE-Q, of 0.45 points), assuming a standard deviation of one for the overall EDE-Q change scores (Hedeker et al., 1999), and also to detect a difference in mean weight gain of one Body Mass Index (BMI) point, which, based on previous research, would be clinically important to detect (Agras et al., 2000; Byrne et al., 2017). Therefore, if a sample loss of approximately 40% is taken into account, the participation of at least 70 participants per group will be required.

### Interventions/Treatment Arms

#### Treatment as Usual

We have chosen not to use a standardized comparison treatment as this would require practical changes in different settings and resource management (including training, supervision and quality control), which will not be feasible. We have therefore chosen to allow centers to follow their own procedures for TAU. We will stratify our analyses by center, which will be adjusted accordingly for any bias.

For TAU inpatient care, the Eating Disorder Unit at the San Juan Hospital will provide a programme using a multidisciplinary team approach (dietician, psychologist, physician and nurse). It will include monitoring of physical risks, nutritional rehabilitation, education on healthy eating and nutrition patterns. Besides it will try to modify/improve thoughts, attitudes, behaviors and feelings that maintain the illness through psychological therapy. Once a patient has stabilized and reached a healthy body mass index, she is discharged. She is then either followed up to assess her progress and to facilitate transition to home or she is referred to a day care center.

Treatment-as-usual in the day care centers (ADANER and CREA) will involve multidisciplinary treatment including dietetic support, psychological interventions, school education, and when appropriate, sessions with psychiatrists. Patients will receive the following psychological interventions: weekly individual cognitive behavioral therapy for eating disorders (CBT-ED); and psychoeducational group therapy on nutrition, emotion management, body image, social skills and problem-solving strategies. Usually, patients attend every day of the week (from Monday to Friday) for an average of 6 h. They receive psychological intervention sessions and they also make the different meals of the day. Parents/carers can also access a fortnightly parent support group.

The TAU-only (control condition) group will have no access to the intervention materials or the ECHOMANTRA intervention sessions. At the end of the study, individuals randomized to the TAU-only condition will be offered the self-help components of the intervention.

#### Treatment-as-Usual Plus Patient and Carer Skills-Sharing Intervention (ECHOMANTRA)

In the experimental group, the adaptation of the ECHOMANTRA programme will be implemented. The contents of the intervention will be translated into the Spanish language and adapted to a Spanish-speaking cultural context.

##### Patients

Based on the interpersonal model of AN, the “MANTRA” part of the ECHOMANTRA intervention addresses patients’ modifiable characteristics, such as difficulties in emotional regulation, interpersonal relationships and eating. The intervention includes a workbook with an emphasis on specific behavioral change strategies. The workbook is organized into eight chapters that correspond to the contents of the eight sessions of the programme.

MANTRA will involved eight, weekly, individual online sessions lasting 60 min, which will be delivered by assistant psychologists trained by the first and principal authors of the study (YQ and MJQ). During the sessions, the trained psychologist will encourage patients to reflect on the information and exercises proposed in the workbook. They will also carry out a series of activities taken from the MANTRA programme to further develop the contents included in the corresponding chapter of the workbook.

Each session will be themed following the structure of the patient workbook. Specifically, the focus of each session will be as follows: the first and second sessions will be on psychoeducation and motivation to change; the third session will be on skills to develop acceptance and self-compassion; the fourth and fifth sessions will be on skills to improve social functioning and to explore thinking styles; the sixth session will be on emotion management; and the seventh and eighth sessions will be on planning for the transition through goal setting, use of social support and implementation intentions.

For a more detailed description of the exercises, see Schmidt et al. (2014).

##### Carers

Carers allocated to the intervention group will receive a carer workbook.

ECHO will involve eight, weekly, individual online sessions, lasting 60 min, which will be delivered by assistant psychologists trained by the first and principal authors of the study (YM and MJS). During the sessions, the trained psychologist will encourage discussion about the information and exercises proposed in the workbook. In addition, for a deeper understanding of the contents of each session presented in the workbook, carers will read and do activities from the book “Skills based caring for a loved one with an eating disorder: The New Maudsley Method” (Treasure et al., 2011). (Spanish version: Los trastornos de la alimentación: guía práctica para cuidar de un ser querido Treasure et al., 2011). These sessions will also include some of the video-clips from the Digital Versatile Disc (DVD) for carers “How to Care for Someone with an Eating Disorder”^1^. This DVD includes practical strategies and techniques to help carers develop skills and knowledge to help their loved one move toward recovery and to look after their own wellbeing by following the “New Maudsley Approach.”

This book and the DVD are designed to help carers develop self-reflective skills in order to develop confidence, compassion and the courage to take risks. Both resources will show them how to experiment with changes in their caring behavior so they can be more helpful to a loved one suffering from an eating disorder.

The workbook and online sessions will provide a skills training programme that includes training in stress management, communication (based on motivational interviewing techniques), strategies to reduce accommodation and expressed emotion and to increase extinction training and new habits at home *via* effective social support.

### Outcomes

#### Patients

This study will consider primary and secondary outcomes measured at the four evaluation points: baseline (T0), post-intervention (T1), 3-month follow-up (T2) and 6-month follow-up (T3).

The following will be assessed as primary measures: ED symptomatology and psychological well-being. Secondary outcomes will include the following: body mass index (BMI), psychosocial adjustment, perfectionism, obsessive-compulsive symptomatology, motivation to change and hospital readmission. All outcomes and the instruments to be used are shown in Table 1.

**TABLE 1:** Outcomes and measures/instruments.

	Outcome	Measure/instrument	References
**PATIENTS**			
1	Body mass index		
2	ED Symptomatology	Eating Disorder Examination (EDE-Q)	Fairburn and Beglin, 1994; Spanish Validation (SV): Peláez-Fernández et al., 2012
3	Eating pattern	Daily food self-reporting	
4	Psychological well-being	Depression Anxiety and Stress Scales (DASS-21)	Lovibond and Lovibond, 1995; SV: Bados et al., 2005
5	Psychosocial adjustment	Eating Disorders Quality of Life (EDQL)	Engel et al., 2006
6	Perfectionism	Child and Adolescent Perfectionism Scale (CAPS)	Flett et al., 1997^1^; SV: Castro et al., 2004
7	Obsessive-compulsive symptoms	Obsessive Compulsive Inventory-Revised (OCI-R)	Foa et al., 2002; SV: González et al., 2011
8	Adherence to treatment	Drop-out rate, number of sessions completed and task completion between sessions.	
9	Motivation to change	Visual analogue scale that assesses confidence and importance in changing symptoms of ED (*ad hoc*)	
10	Admission	Number of readmissions to hospital during the intervention and follow-up periods. Record medical history	
11	Patient feedback form	Patients’ satisfaction and experiences in the study.	
**CARERS**			
1	Psychological well-being	Depression Anxiety and Stress Scales (DASS-21)	Lovibond and Lovibond, 1995; SV: Bados et al., 2005
2	Expressed emotion	Family Questionnaire	Wiedemann et al., 2002; SV: Sepúlveda et al., 2014
3	Burden	Eating Disorders Symptom Impact Scale (EDSIS-S).	Sepúlveda et al., 2008; SV: Carral-Fernández et al., 2013
4	Accommodation to illness	Accommodation to Illness Symptoms Scale (AESED).	Sepúlveda et al., 2009; Quiles Marcos et al., 2016
5	Care skills	Caregiver skills scale.	Hibbs et al., 2015b; SV: Vintró-Alcaraz et al., 2018
6	Parents’ perception of efficacy	Parents vs anorexia.	Rhodes et al., 2005
7	Carer feedback form	Carers’ satisfaction and experiences in the study.	

*^1^Flett, G. L., Hewitt, P. L., Boucher, D. J., Davidson, L. A. and Munro, Y. (1997). The child-adolescent perfectionism scale: development, validation, and association with adjustment. Unpublished manuscript.*

##### Clinical Assessment

The health care providers at the day care center or the 24-h hospital unit will submit the following information on the patient: BMI from monthly clinical measurement and up to 9 months post-randomization, diagnosis, age at onset of disorder, evolution of illness over time (duration), admissions prior to current one, comorbidity and readmission after being discharged.

#### Carers

For carers the following outcome measures will be considered: emotional state, expressed emotion, impact from and accommodation to eating symptoms, coping skills, and parents’ perceived efficacy in dealing with the ED (see Table 1).

Patients and carers in the ECHOMANTRA-plus- TAU (treatment) arm will complete a “Participant Feedback Form.” It is a self-report measure created *ad hoc* for completion at the end of the intervention. It will assess participants’ experiences and satisfaction with the study. They will be asked to provide their views regarding: what they found beneficial and/or challenging, what they enjoyed and/or did not like, the transferability of ECHOMANTRA skills to their routine, and their suggestions for further improvements to the intervention.

### Blinding

Given the nature of the study design, all participants and therapists will be aware of the treatment condition.

The person responsible for creating the sequence of randomization will not belong to the research team and will not have other role in this research project. The researcher responsible for making the analyses will be a specialist in statistical methodology and only will participate in this aspect of the study. The research assistant who administers the assessment at each time period will not deliver the intervention. The statistician will be blinded to the condition allocated to the patient and their carer.

### Statistical Analysis

Firstly, a covariance analysis (ANCOVA) will be carried out using the pre-test scores as a covariate to analyze the impact of the combined intervention. Secondly, a repeated measures analysis of variance will be used to analyze the short and medium-term efficacy of the combined intervention in comparison to the usual intervention between and within groups at different time points, and effect size values will also be considered. Stratification will be performed in the analyses by the admission center and “diagnosis” variable will be controlled. IBM SPSS Statistics 24.0 will be used for all the analyses (IBM Corp, 2016).

## Discussion

The treatment of eating disorders should include patients and carers in order to improve patients’ outcomes and adherence (Treasure and Nazar, 2016). In this sense, ECHOMANTRA is an intervention that can improve outcomes during and following intensive care for adolescents with AN (Cardi et al., 2017; Adamson et al., 2019). ECHOMANTRA should reduce patients’ distress and eating disorder symptoms. For carers, outcomes have been related to a reduction in distress and an improvement in their skills.

ECHOMANTRA is protocolized in eight sessions, so it can be replicated in different contexts, such as a day hospital or inpatient unit. It was designed to be affordable, scalable, and to potentially have a wide reach (Cardi et al., 2017). Treatment sessions will be developed online, which will allow both patients and family members to adapt their learning and improvement in therapeutic skills to their daily routine in order to increase engagement. This design will make it possible to overcome some of the obstacles that make it difficult for participants to adhere, especially carers.

This is a multi-center trial, which will take place in two different types of services (day hospital and inpatient unit). It will allow us to evaluate intervention effectiveness according to the therapeutic context and its impact on the generalizability of the data. Also, MANTRA was originally developed for adult AN patients. This study will examine the usefulness of the MANTRA treatment programme for adolescents with other EDs and not just AN, thereby enhancing current knowledge about potential treatments for these patients.

This trial has some limitations. Firstly, two questionnaires that will be used in this RCT, the EDQL and the “Parents vs Anorexia Questionnaire” are not validated in the Spanish population. As a result, our team will validate them. Another limitation is that not only AN patients will receive the MANTRA protocol, but it will also be received by BN and those with OSFED. Another limitation is the fact that we have translated all the original protocols into Spanish, and we will use them without a previous pilot study. Another limitation is the possible difficulties for both members of the dyad (patient and carer) to be involved in the intervention during all sessions. To facilitate the adherence of both, the schedule for each of the sessions will be agreed on individually with each of the participants. Finally, special attention will be paid to possible difficulties in maintaining adherence in the control group. To this end, they will be offered the self-help components of the intervention at the end of the study.

The strengths of this study will be the randomized control study design and protocolized therapist guidance during the intervention. Moreover, individualized interventions for patients and carers reinforce trial soundness.

Clinical implications will be related to improving psychological treatment for ED disorders. In our opinion, findings from the ECHOMANTRA trial will be able to optimize inpatient/day-patient treatment and improve our knowledge about the factors that maintain the illness for those with a severe and enduring ED. An additional benefit could be found in the assessment of MANTRA’s effectiveness for BN and OSFED and not only for AN.

Finally, this paper outlines the protocol for a study that should improve treatment in ED patients. We have outlined the components of the ECHOMANTRA intervention and have clearly stated the research methodology as recommended in CONSORT 2010 guidelines (Schulz et al., 2010).

## Data Availability Statement

The raw data supporting the conclusions of this article will be made available by the authors, without undue reservation.

## Ethics Statement

The studies involving human participants were reviewed and approved by Ethics Committee of University Hospital of San Juan of Alicante, and Ethics Committee of University Miguel Hernández of Elche. Written informed consent to participate in this study was provided by the participants’ legal guardian/next of kin.

## Author Contributions

YQ spearheaded the design of the trial protocol and development of intervention materials in collaboration with MQ, EL, MR, ÁR, ME, CR, and VE. All authors contributed to the article and approved the submitted version.

## Conflict of Interest

The authors declare that the research was conducted in the absence of any commercial or financial relationships that could be construed as a potential conflict of interest.

## Publisher’s Note

All claims expressed in this article are solely those of the authors and do not necessarily represent those of their affiliated organizations, or those of the publisher, the editors and the reviewers. Any product that may be evaluated in this article, or claim that may be made by its manufacturer, is not guaranteed or endorsed by the publisher.

## Footnotes

1
http://www.succeedfoundation.org/work/pre_order_dvd
